# A System of Obstetrics

**Published:** 1889-08

**Authors:** 


A System of Obstetrics by American Authors, edited by Barton Cook Hurst, M. D., Associate Professor of Obstetrics in the University of Pennsylvania, Obstetrician to the Philadelphia and Maternity Hospitals; Gynecologist to the Orthopædic Hospital; Fellow of the College of Physicians of Philadelphia. Vol. II. illustrated with two hundred and twenty-one engravings on wood. Philadelphia, Lea Brothers & Co., 1889. 854 large oc. pages elegantly and substantially bound, and with that typographical neatness and accuracy for which Lea Brothers are noted.

In the present volume there is ably presented the following important subjects:

Diseases and Accidents of Labor, by Theophilus Pavin, M. D., LL. D.

The Forceps—Embryotomy, by Edward P. Davis, A. M., M. D.

The Primitive Induction of Labor, by James C. Cameron, M. D.

The Cæsarean Operation, Symphysiotomy, Laparotomy, etc., by Robt. P. Harris, A. M., M. D.

Puerperal Infection, by Henry J. Garrigues, A. M., M. D.

Inflammation of the Breasts and allied diseases connected with childbirth, by Henry J. Garrigues, A. M., M. D.

The Etiology of Puerperal Fever, by Harold C. Ernst, M. D.

Some Complications of the Puerperal State independent of septic infection by Barton Cook Hirst, M. D.

Insanity and Diseases of the nervous system in the child-bearing woman, by James H. Lloyd, A. M., M. D.

The management, and the diseases of the new-born infant, by J. Lewis Smith, M. D.

The surgical disease of infancy and early childhood, by Stephen Smith, A. M., M. D.

Congenital anomolies of the eye, by G. E. DeSchweintz, M. D.

The character of the authors of the foregoing articles is sufficient guarantee of the ability with which they are presented. The book should be in the library of every progressive medical man.

